# Cells deficient in base-excision repair reveal cancer hallmarks originating from adjustments to genetic instability

**DOI:** 10.1093/nar/gkv222

**Published:** 2015-03-23

**Authors:** Enni Markkanen, Roman Fischer, Marina Ledentcova, Benedikt M. Kessler, Grigory L. Dianov

**Affiliations:** 1 Cancer Research UK and Medical Research Council Oxford Institute for Radiation Oncology, Department of Oncology, University of Oxford, Roosevelt Drive, Oxford OX3 7DQ, United Kingdom; 2 Target Discovery Institute, Nuffield Department of Medicine, University of Oxford, Oxford OX3 7FZ, United Kingdom; 3 Institute of Cytology and Genetics, Siberian Branch of the Russian Academy of Sciences, Lavrenteva 10, 630090 Novosibirsk, Russia

## Abstract

Genetic instability, provoked by exogenous mutagens, is well linked to initiation of cancer. However, even in unstressed cells, DNA undergoes a plethora of spontaneous alterations provoked by its inherent chemical instability and the intracellular milieu. Base excision repair (BER) is the major cellular pathway responsible for repair of these lesions, and as deficiency in BER activity results in DNA damage it has been proposed that it may trigger the development of sporadic cancers. Nevertheless, experimental evidence for this model remains inconsistent and elusive. Here, we performed a proteomic analysis of BER deficient human cells using stable isotope labelling with amino acids in cell culture (SILAC), and demonstrate that BER deficiency, which induces genetic instability, results in dramatic changes in gene expression, resembling changes found in many cancers. We observed profound alterations in tissue homeostasis, serine biosynthesis, and one-carbon- and amino acid metabolism, all of which have been identified as cancer cell ‘hallmarks’. For the first time, this study describes gene expression changes characteristic for cells deficient in repair of endogenous DNA lesions by BER. These expression changes resemble those observed in cancer cells, suggesting that genetically unstable BER deficient cells may be a source of pre-cancerous cells.

## INTRODUCTION

The molecular origin of all cancers lies in mutations in a cell's DNA sequence (1). Indeed, genomic instability and mutation is recognised as one of the very few enabling characteristics that clearly drive tumorigenesis by facilitating the acquisition of all other core hallmarks of cancer (2). Mutations can arise when the integrity of DNA is challenged by various agents deriving from exogenous sources (3). To counteract the formation of mutations, cells have evolved a plethora of DNA repair pathways that sense, report and correct alterations in DNA (4). Deficiencies in some of these repair pathways have been implicated to play a key role in the induction and progression of cancer (5,6). However, even without damage deriving from exogenous sources (such as UV irradiation or tobacco smoke), DNA is prone to spontaneous alterations caused by its chemical instability and many different intracellular (endogenous) mutagens (reviewed in (3)). It is estimated that every single cell acquires as many as 10 000–20 000 lesions per day under physiological and unstressed conditions (7). The major cellular pathway to safeguard the genome against these frequent and constantly arising DNA lesions is base excision repair (BER), which is responsible for repairing a multitude of different base alterations and DNA single strand breaks (SSBs) (8). The importance of BER for cellular maintenance is exemplified by the fact that knocking out any of the core BER pathway genes is embryonically lethal (8). BER is initiated by damage-specific DNA glycosylases, which identify and release the corrupted base by hydrolysis of the N-glycosylic bond linking the DNA base to the sugar phosphate backbone (reviewed in (9)). In mammalian cells, the arising abasic site (AP-site) is further processed by AP-endonuclease 1 (APE1), which cleaves the phosphodiester bond 5′ to the AP-site, generating a SSB with a 5′-sugar phosphate. Further processing of this intermediate is carried out by a DNA repair complex that includes DNA polymerase β (Pol β), XRCC1 and DNA ligase IIIa (Lig III). Pol β possesses a dRP- lyase activity that removes the 5′-sugar phosphate and also, functioning as a DNA polymerase, adds one nucleotide to the 3′-end of the arising single-nucleotide gap. Finally, the XRCC1-Lig III complex seals the DNA ends, therefore accomplishing DNA repair (8). XRCC1 is a scaffold protein that is absolutely required for formation and stabilisation of the ternary Pol β-XRCC1-Lig III complex on SSBs arising spontaneously or generated by APE1 during BER (10,11). Hence, cells deficient in XRCC1 are characterised by reduced DNA repair and genomic instability (reviewed in (12)).

While the role of faulty DNA double-strand break repair in tumorigenesis is widely accepted, the role of other DNA repair pathways, and in particular the contribution of BER deficiency to cell transformation is far from being understood. Given the importance of BER to cellular DNA homeostasis, it is conceivable that a deficiency in this pathway could contribute to carcinogenesis, or even initiate very early steps of cellular transformation by acting as enabler of carcinogenesis through induction of genomic instability without the administration of exogenous damaging agents. Indeed, knockdown of XRCC1 has been shown to cause a deficiency in repair of DNA base damage and lead to an accumulation of unrepaired SSBs (12). Furthermore, haploinsufficiency in XRCC1 was found to sensitise mice toward treatments with carcinogenic substances, resulting in enhanced formation of precancerous lesions (13). Thus, we hypothesised that a deficiency in BER and the following accumulation of unrepaired SSBs (that derive from endogenous, spontaneously occurring DNA damage) would not only lead to genetic instability and accumulation of mutations, but also lead to specific changes in protein expression as reaction to this permanent genetic instability. Moreover, since genetically unstable cells have an increased probability to become transformed, we predicted that this ‘imprinting’ of changes on the proteomic level induced by genetic instability might later be found in pre-malignant and transformed cells, thus underscoring the role of BER deficiency in tumorigenesis. To test this hypothesis, we performed a proteomic analysis using stable isotope labelling of amino acids in cell culture (SILAC) in primary human cells with a BER deficiency brought about by knocking down XRCC1 using siRNA. Analysis of the most significant changes revealed profound alterations in cellular serine biosynthesis, the one-carbon metabolism, amino acid uptake and synthesis as well as regulation of cellular redox status. We also detected significant changes in expression of proteins regulating tissue homeostasis, in particular proteins involved in cellular motility, tissue remodelling and cellular invasion. All these changes point to metabolic reprogramming as well as alterations in tissue invasion and metastasis, each of which is considered as a hallmark of cancer cells (2), suggesting that these changes may originate from genetically unstable BER deficient cells. Additionally, by shedding light on the role of BER deficiency in creating preconditions for cell transformation, our observations also suggest that these observed changes in metabolism and cellular motility are not late-onset adaptations in full-blown cancerous cells, as is often assumed, but rather denote early adjustments to genetic instability that can be found later in transformed cells.

## MATERIALS AND METHODS

### Cell culture

Human primary fibroblasts (TIG-1) were purchased from Coriell and cultured under standard conditions in Dulbecco's modified Eagle's medium (DMEM) (Invitrogen, 21885-108) supplemented with 15% Fetal Calf Serum (FCS). For SILAC experiments, cells were grown in light arginine/lysine (R0K0) or DMEM supplemented with heavy arginine [^13^C_6_ Arg] and lysine [D_4_ Lys] (R6K4) labeled DMEM (Dundee cell products Ltd, LM014 and LM016) and 15% dialysed FCS (molecular weight cut-off 3.5 K, Thermo Pierce) for a minimum of seven population doublings to ensure optimal labeling efficiency.

### Mass spectrometry

Cell fractionation for MS/MS analysis. Freshly harvested cells were resuspended in two packed cell volumes of cold buffer A (20 mM Tris pH 7.4, 2.5 mM MgCl_2_, 0.5% (v/v) NP-40, 1 mM *N*-ethylmaleimide (NEM), 1 μg/ml Leupeptin, Pepstatin, Chymostatin, Aprotinin and Phenylmethanesulfonyl fluoride (PMSF)), vortexed to resuspend, and incubated for 10 min on ice. Nuclei were pelleted by centrifugation of extracts at 10 000 rpm/10 621 rcf for 2 min. The supernatant (cytoplasmic fraction) was transferred to a fresh tube and stored on ice. The nuclear cell pellet was then resuspended in buffer B (20 mM phosphate buffer pH 8.0, 500 mM NaCl, 1 mM EDTA, 0.75% (v/v) Triton X-100, 10% glycerol, 5 mM MgCl_2_, 1 mM NEM, 1 μg/ml leupeptin, pepstatin, chymostatin, aprotinin and PMSF) in the same volume as used before for buffer A, vortexed to resuspend, and incubated for 10 min on ice. Insoluble components were then removed by spinning 2 min at 10 000 rpm/10 621 rcf, and the supernatant (nuclear fraction) was transferred to a fresh tube. Protein samples were then reduced, alkylated and precipitated by chloroform/methanol precipitation (14). The precipitate was resuspended in 6 M urea in 100 mM Tris pH 7.4, digested with trypsin, desalted using C18 Sep Pak column cartridges and dried *in vacuo*. Samples were resuspended in buffer A (98% H_2_O, 2% acetonitrile, 0.1% formic acid) and subjected to analysis by nano- ultraperformance liquid chromatography tandem mass spectrometry nUPLC-MS/MS as described (14). In brief, we used a nUPLC system (Water nAcquity) with a 75 μm x 250 mm, 1.7 μm particle size) analysis coupled to a Thermo LTQ Orbitrap Velos (60 000 resolution, Top 20, CID) workflow and a gradient of 1–40% acetonitrile in 120 min at a flow rate of 250 nl/min. The analysis of SILAC MS data was analysed using MaxQuant software as described previously (15). Pathway analysis of the SILAC data was generated through the use of IPA (Ingenuity^®^ Systems, www.ingenuity.com).

### Western blotting and antibodies

Whole cell extracts for Western blotting were prepared as described previously (16). Proteins were separated by sodium dodecyl sulphate-polyacrylamide gel electrophoresis (SDS-PAGE) and transferred onto Immobilon-FL Polyvinylidene fluoride (PVDF) membranes (Millipore) according to standard procedures (Novex). Blots were probed with following antibodies: XRCC1 (Neomarkers, MS-1393-P0), DNA polymerase β (raised in-house and affinity-purified), α-tubulin (Sigma, T6199), MCM4 (abcam, ab4459-50), RP-A p32 (Bethyl, A300-244A), PCNA (Santa Cruz, sc-56), α-actin (Abcam, ab6276), PARP-1 (raised in-house and affinity-purified), p21 (Cell signaling, 12D1), PSAT1 (Novusbio, 21020002), PHGDH (Sigma, HPA021241), p53 (Santa Cruz, sc-126), PNKP (Abnova, H00011284-B01). Secondary antibodies conjugated with Alexa Fluor 680 (Molecular Probes) and IRDye^®^ 800 (Rockland) fluorescent dyes were used. Detection and quantification was carried out using an Odyssey image analysis system (Li-Cor Biosciences).

### Knockdown by siRNA

Transfections with siRNA were carried out using Lipofectamine RNAiMAX reagent (Invitrogen) according to the manufacturer's protocol. Cells were analysed at indicated time-points after transfection. siRNA sequences (Eurogentec) were as following: XRCC1 sequence 1: 5′-AGGGAAGAGGAAGUUGGAU-3′, XRCC1 sequence no. 2: 5′-GGAAGAUAUAGACAUUGAG-3′, PNKP: 5′-CACACUGUAUUUGGUCAAU-3′, TDP1: 5′-CUAGACAGUUUCAAAGUG-3′ (17), ATF4 siRNA (pool of 4 siRNAs, Dharmacon) was a kind gift from Christos Zois and Adrian Harris (Oxford). Control transfections were carried out without addition of siRNA.

### Quantitative RT-PCR

Total RNA was purified using the RNeasy kit (Qiagen) and cDNA was prepared using the SuperScript RT-PCR system (Invitrogen). Quantitative RT-PCR was performed using Absolute Blue QPCR SYBR low ROX Mix (Thermo Scientific) according to the manufacturer's protocol. Reactions were carried out in duplicate for each target transcript using a 7500 Fast Real-Time PCR System (Applied Biosystems). The comparative CT method was applied for quantification of gene expression, and values were normalised against Glyceraldehyde 3-phosphate dehydrogenase (GAPDH) as control. Results were expressed as fold change in mRNA levels. Following primers were used: PHGDH for (5′-ATCTCTCACGGGGGTTGTG-3′), PHGDH rev (5′-AGGCTCGCATCAGTGTCC3′), PSAT1 for (5′-CGGTCCTGGAATACAAGGTG3′), PSAT1 rev (5′- AACCAAGCCCATGACGTAGA-3′), ATF4 for (5′-GGGACAGATTGGATGTTGGAGA-3′), ATF4 rev (5′-ACCCAACAGGGCATCCAAGT-3′), MTHFD2 for (5′-TGCTGCAGGTATTCCAAATC-3′), MTHFD2 rev (5′-GGGTTTGGCAGTTACAGGAT-3′), ATF4 for (5′-GGGACAGATTGGATGTTGGAGA-3′), ATF4 rev (5′-ACCCAACAGGGCATCCAAGT-3′), GAPDH for (5′-AGCCACATCGCTCAGACAC-3′), GAPDH rev (5′-GCCCAATACGACCAAATCC-3′), TDP1 for (5′-ATGGTGATAAGCGAGAGGCT-3′), TDP1 rev (5′-CGGAGGCCTTCTTCATAGAG-3′).

### G1 arrest

Cells were grown until confluence, after which KD was performed with siRNA as usual.

### Intracellular GSH measurement

Measurement of GSH levels in living cells was performed after (18). In short, cells were seeded into 24-well plates at the indicated densities 48 h after KD. Twenty four hours later intracellular GSH was labeled by Dibromobimane, which is fluorescently activated upon conjugation with thiol groups. The signal was normalized to the cell density as assessed by Hoechst 33342 staining. Fluorescent intensities were read with a plate reader (EnVision, Perkin Elmer).

### Graphical display of results and statistical analysis

For all statistical analysis and graphical display, the program GraphPad Prism (www.graphpad.com) was used.

### Flow cytometry

For FACS, trypsinized cells were fixed in ice-cold 70% ethanol for at least 30 min. After removal of the fixation solution by centrifugation, cells were treated with 100 μg/ml of DNase free RNase A at 37ºC in phosphate buffered saline for 30 min (Sigma) and further stained with 10 μg/ml propidium iodide (Sigma). Samples were run on a Becton–Dickinson FACScan (BD Biosciences) and the cell cycle distribution analysed using Modfit LT software (Verity Software House).

### Alkaline comet assay

The comet assay was performed as recently described (10). Briefly, cells were harvested by trypsinisation, diluted to a concentration of 2 × 10^5^ cells/ml in medium, and embedded on a microscope slide in 1% low-melting agarose in phosphate buffered saline (PBS) (Bio-Rad). The slides were lysed in buffer containing 2.5 M NaCl, 100 mM ethylenediaminetetraacetic acid (EDTA), 10 mM Tris–HCl pH 10.5, 1% (v/v) Dimethyl sulfoxide (DMSO) and 1% (v/v) Triton X-100 for 1 h at 4°C. Subsequently, the slides were then incubated in the dark for 30 min in cold electrophoresis buffer (300 mM NaOH, 1 mM EDTA, 1% (v/v) DMSO, pH >13) to allow DNA unwinding prior to electrophoresis at 25 V, 300 mA for 25 min. Neutralisation of the slides was performed with 0.5 M Tris–HCl (pH 8.0). The slides were stained with SYBR Gold (Invitrogen) and analysed using the Komet 5.5 image analysis software (Andor Technology, Belfast, Northern Ireland).

## RESULTS

### Outline of experimental approach

**Figure 1.**
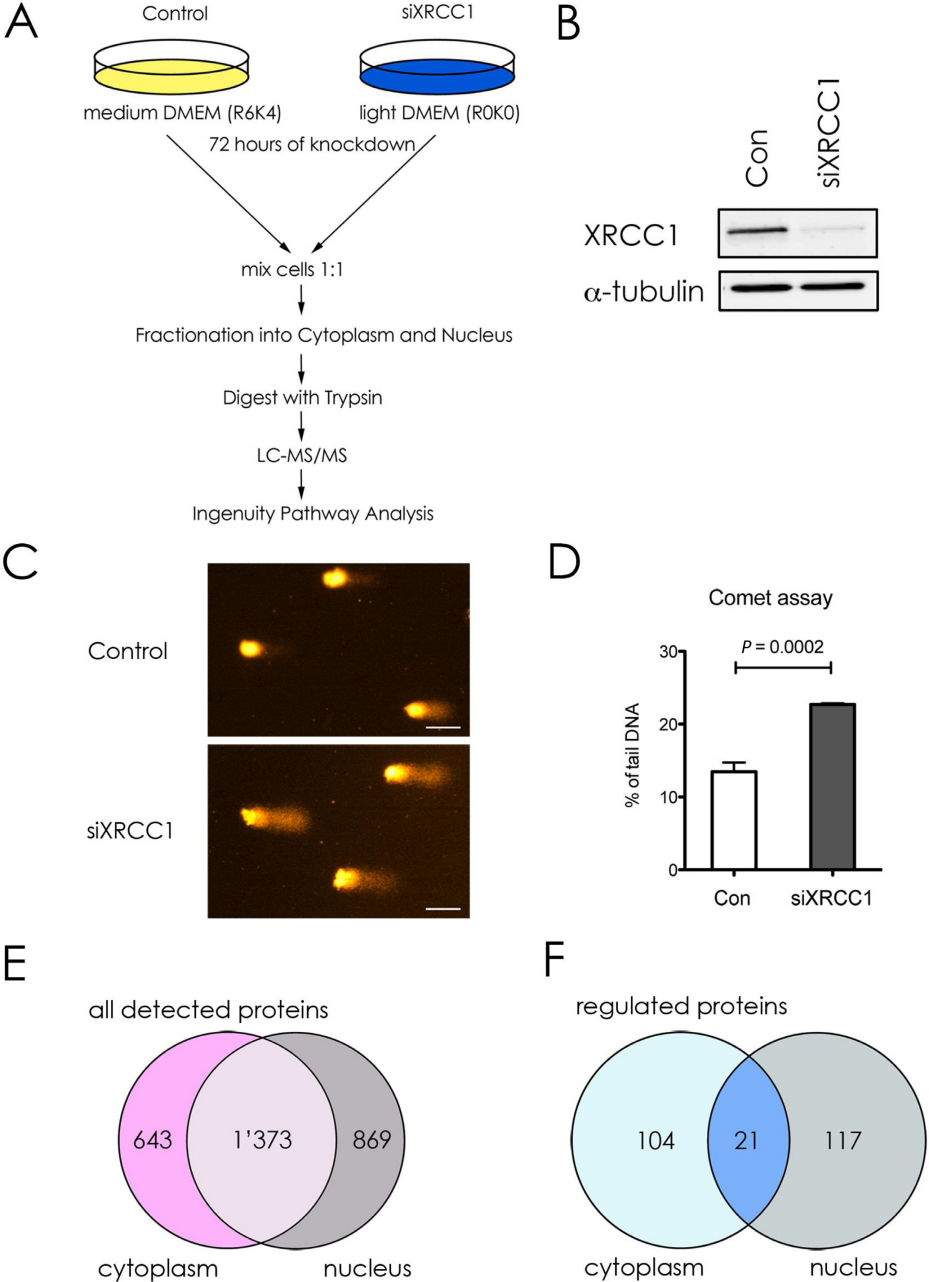
Outline and validation of the experimental approach. (**A**) Workflow scheme for the SILAC analysis. siRNA-mediated knockdown of XRCC1 or control was performed with Tig-1 cells grown in light (R0K0) or medium (R6K4) containing DMEM for 72 hours. Cells were harvested, mixed at a ratio 1:1 and fractionated into cytoplasmic and nuclear fractions. Precipitated proteins were digested with trypsin and analyzed via liquid chromatography MS/MS to quantify differences in peptide abundance. Results were further analyzed using the Ingenuity Pathway Analysis (IPA). (**B**) Western blot analysis of the XRCC1 levels in WCE made from cells used in (**A**). α-tubulin was used as loading control. (**C**) Representative Comets for Alkaline Comet assay of siXRCC1 and control cells. Scale bar = 50 μm. (**D**) Quantification of three independent Alkaline Comet assay experiments. Values are mean ± SD. The *P*-value was calculated using Student's two-tailed *t*-test. **(E** and **F)** Venn diagrams showing all detected proteins (E) or proteins up- or downregulated ≥2-fold (F) upon XRCC1 KD, as detected by IPA.

To perform quantitative SILAC analysis, primary human BER deficient or control cells were cultured in DMEM with light R0K0 or medium R6K4 labeled isotopes, respectively (Figure 1A). A deficiency in BER was brought about by knocking down (KD) the BER scaffold protein XRCC1 by siRNA transfection. Seventy-two hours after knockdown cells were harvested, and a fraction was set aside to prepare whole cell extracts (WCE) for validation purposes. The remaining cells were combined at a 1:1 ratio and, in order to increase the resolution for enhanced detection of peptides by MS/MS analysis, fractionated into cytoplasmic and nuclear fractions. Thereafter, cellular proteins were extracted, digested with trypsin, desalted and subjected to analysis by quantitative mass spectrometry (MS/MS). MS Data were extracted, and protein identification and SILAC based quantitation were performed using the MS/MS and MS data sets, respectively (see ‘Material and Methods' section). Relative quantitation reflecting changes in protein abundance ratio are summarized in Supplementary Table S2.

### XRCC1 knockdown leads to DNA damage accumulation and dysregulation of gene expression

To verify the KD efficiency of XRCC1, we performed western blot analysis on WCE (Figure 1B). We observed a decrease of about 80% in XRCC1 expression. Importantly, a similar decrease in XRCC1 levels was also observed in the SILAC dataset from the MS/MS analysis (2.7-fold decrease, Table 2, and Supplementary Table S2). The accumulation of SSBs after XRCC1 knockdown depends on spontaneous formation of DNA base lesions. During attempted repair some of these lesions are converted into SSBs due to the deficiency in DNA gap-filling (by Pol β) and ligation (by DNA ligase III). This means that the rate-limiting step is accumulation of endogenous lesions, which is followed by SSB accumulation. As we have recently demonstrated (16) that significant accumulation of DNA single strand breaks is achieved at 72 h after XRCC1 siRNA application, we thus chose this time-point for further investigation. The partial deficiency in DNA repair caused by XRCC1 KD resulted in the persistent accumulation of unrepaired endogenous DNA lesions at 72 h after KD of XRCC1, as measured by the alkaline Comet assay which detects DNA strand breaks and alkaline labile base lesions (Figure 1C and D) (19). Of note, the extent of XRCC1 KD does not completely abrogate the ability to repair SSBs, but decreased the BER capacity, which led to a marked delay in, but not a complete absence of SSB repair (20).

**Table 1.**
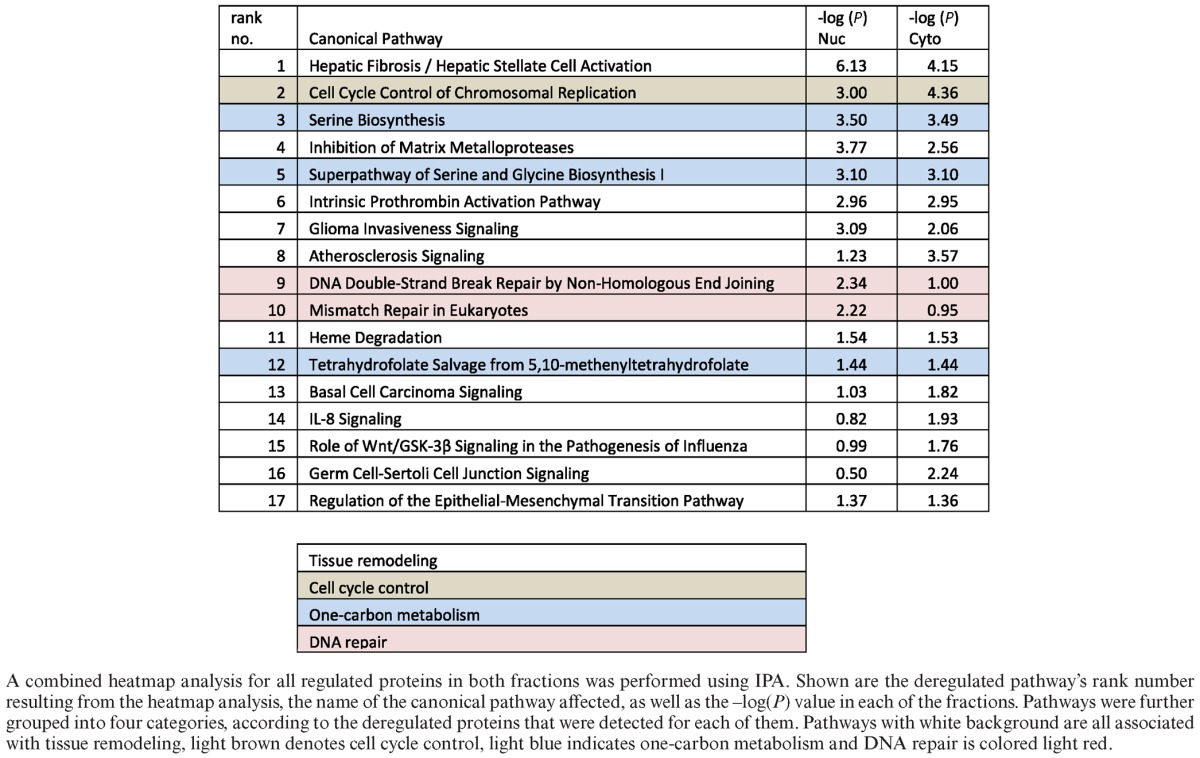
The top regulated pathways upon XRCC1 knockdown

**Table 2.**
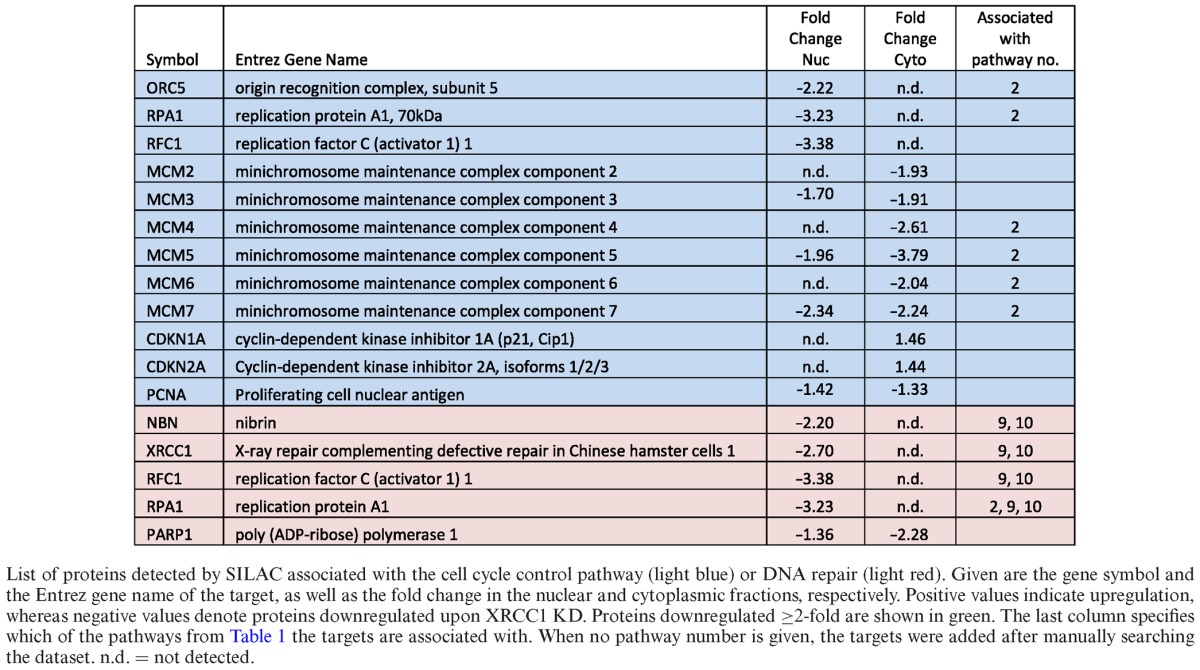
Proteins associated with cell cycle control and DNA repair that are deregulated upon XRCC1 knockdown

A total of 2885 proteins were identified in the sample by mass spectrometry, of which 643 were uniquely found in the cytoplasmic, and 869 uniquely in the nuclear fraction, respectively (Figure 1E and Supplementary Table S2). 1373 proteins were identified in both fractions. Applying a cutoff value of a minimum 2-fold change in abundance upon XRCC1 KD revealed 242 proteins significantly up- or downregulated, 104 of which were in the cytoplasm, 117 in the nucleus and 21 proteins present in both fractions (Figure 1F).

### XRCC1 knockdown activates the G_1_/S checkpoint

Further analysis of the peptides identified by MS/MS was performed using the Ingenuity pathway analysis (IPA) software, which allows the identification and ranking of deregulated pathways in a specific dataset by extrapolating from to changes in abundance of individual proteins involved in these pathways. Using this method, by combining proteomic data from both the nuclear and cytoplasmic fractions in a so-called combined heatmap analysis, a list of most deregulated (up- or downregulated) pathways from the entire dataset was obtained, of which we decided to look closer at the top 17 deregulated pathways (Table 1).

**Figure 2.**
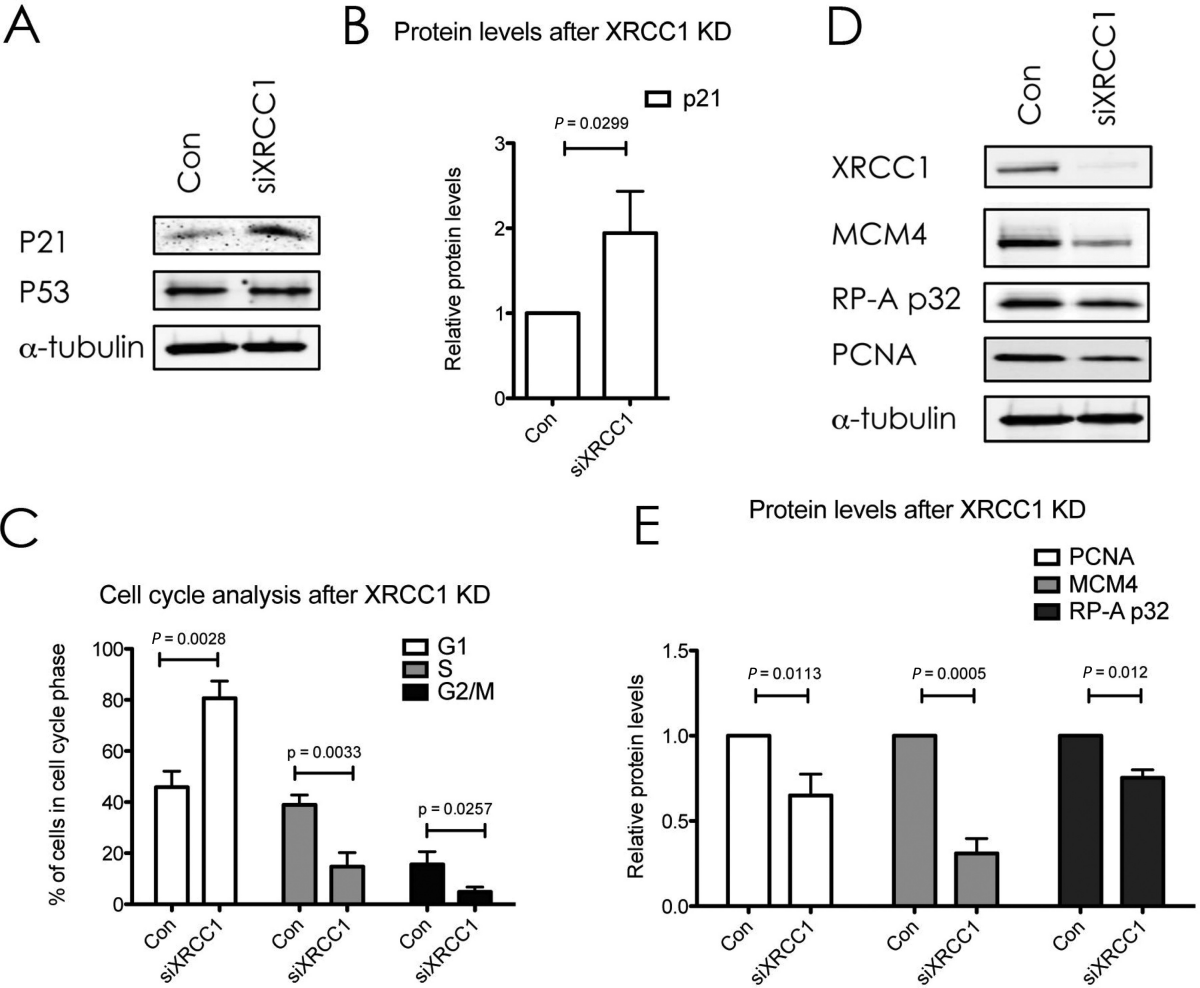
XRCC1 knockdown activates a DNA damage response and delays cell cycle progression. (**A** and **D**) Western blot analysis of the indicated proteins upon XRCC1 KD. α-Tubulin served as a loading control. (**B**) Quantifications of p21 protein levels as seen in (**A**). Values are mean ± SD from four independent experiments, normalized to the loading control. The *P*-value was calculated using Student's *t*-test. (**C**) FACS analysis of cell-cycle distribution after XRCC1 KD. Shown is the percentage of cells in the respective cell cycle phases after control treatment or XRCC1 KD. Values are mean ± SD from three independent experiments. The *P*-value was calculated using Student's two-tailed *t*-test. (**E**) Quantification of protein levels as seen in (**D**). Values are mean ± SD from four independent experiments, normalized to the loading control. The *P*-value was calculated using Student's *t*-test.

As primary cells harbor intact DNA damage signaling pathways, the accumulating DNA damage caused by XRCC1 KD was expected to induce a DNA damage-dependent checkpoint of the cell cycle. Indeed, pathway analysis of the proteomic data by IPA combined heatmap analysis identified an activation of the G_1_/S DNA damage checkpoint, which was highly ranked among the most significantly deregulated pathways in the dataset (Table 1, rank 2). In accordance with this, we observed the induction of the known G_1_ arrest mediators CDKN1A/p16, and CDKN2A/p21, in the MS/MS data (albeit with an induction <2-fold, Table 2). Up-regulation of p21 was confirmed by western blot analysis (Figure 2A and B). Though the G1/S arrest is known to be regulated by p53, we did not observe a change in p53 protein levels upon XRCC1 KD, suggesting that it was activated rather by posttranslational modifications than MDM2 inhibition (Figure 2A). Delay of DNA replication and accumulation of cells in G_1_ phase of the cell cycle were further verified by FACS analysis (Figure 2C). Importantly, however, despite the accumulation of cells in G_1_, these cells were still cycling, as evidenced by the fraction of cells in S and G_2_ phases, suggesting that accumulating SSBs caused by a decrease in the BER capacity induce a transient G_1_ delay to repair the damage before entering S-phase. Additionally, the transient accumulation of cells in G_1_ due to DNA damage led to a clear downregulation of the subunits of the replicative DNA helicase MCM_2–7_, Orc5 (a member of the origin recognition complex proteins), RPA subunit 1 (an important DNA-binding factor at replication forks), PCNA (the ring-shaped molecule that coordinates replication proteins at the replication fork), and RFC subunit 1 (responsible for loading PCNA onto the fork) (Table 2 and Figure 2D and E). Taken together, these data confirmed that the XRCC1 knockdown led to accumulation of DNA lesions, which resulted in a DNA damage-dependent G1/S checkpoint activation. The decrease in S-phase cells also explains, why DNA repair pathways flagged up as significantly changed among the other pathways (Table 1, ranks 9 and 10, and Table 2, light red). Most of these proteins that play a role in DNA repair are also strongly associated with DNA replication (RPA, PCNA, RFC). Therefore, in our dataset, we interpret changes in these DNA repair pathways to be mainly a secondary effect deriving from the G_1_/S-arrest.

### Detailed analysis of the top regulated pathways and proteins

For a detailed assessment of a possible link of BER to pre-malignant cellular transformation, we further analysed the 17 top deregulated pathways obtained by combined heatmap analysis using IPA (see above) (Table 1). Despite a wide diversity of these top pathways at first glance, a high amount of overlapping protein components could be identified between many of them. For example, alpha-2-macroglobulin (A2M) is attributed to pathways no. 1, 4 and 16 (Table 3, last column), whereas matrix metallopeptidase 2 (MMP2) is associated with pathways no. 1, 4, 7, 14 and 17 (Table 3, last column). Taking into account these redundancies in detected targets, these top 17 pathways could be further grouped into four distinct categories summarized in Table 1: tissue remodeling (white), cell cycle control (light brown), one-carbon metabolism (light blue), and DNA repair (light red). To further understand the changes, these categories were analysed in detail as discussed below (except for the cell cycle control pathway, as discussed above). Furthermore, other proteins that are closely related to these top 17 pathways, which however did not score among the top 17 pathways, but displayed significant changes (i.e. changes ≥2-fold up or down), were manually included in Table 3 to enhance the picture of the global changes in these cells.

**Table 3.**
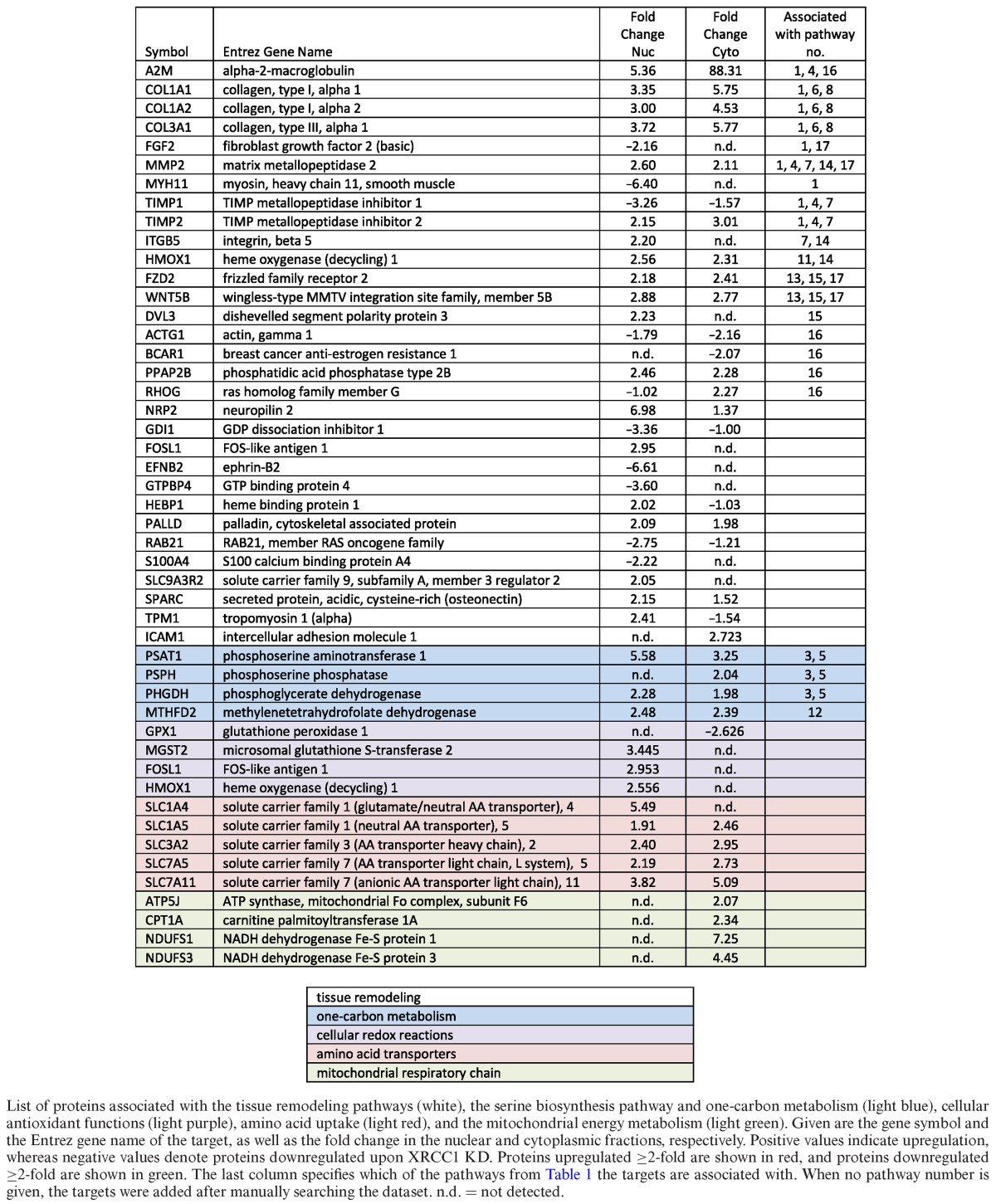
Proteins associated with tissue remodeling, one-carbon metabolism, cellular redox reactions, amino acid transporters and the mitochondrial respiratory chain that are deregulated upon XRCC1 knockdown

### XRCC1 KD induces changes in the one-carbon metabolism, energy metabolism and amino acid synthesis and uptake

**Figure 3.**
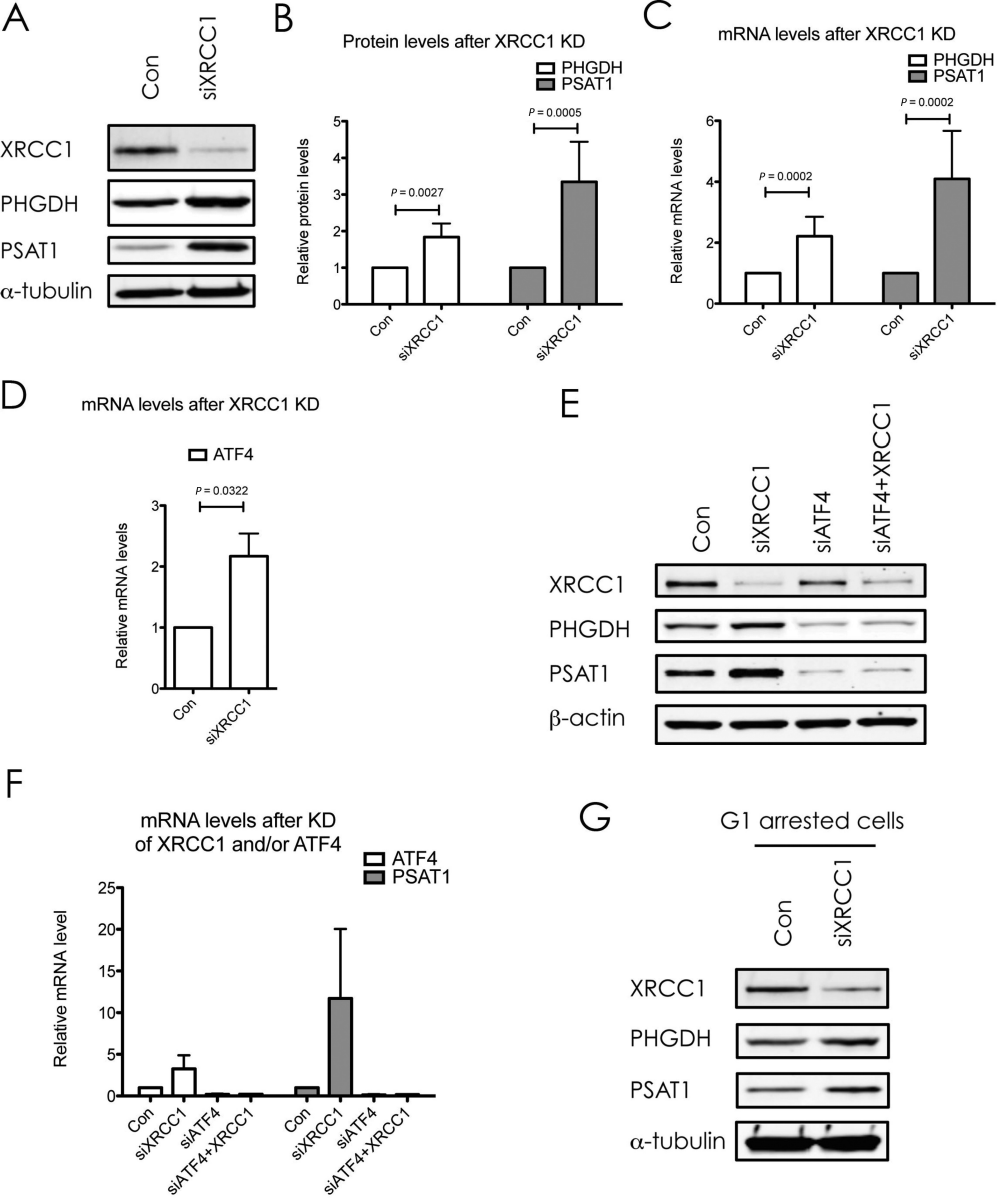
XRCC1 knockdown induces changes in proteins associated with serine biosynthesis, the one-carbon metabolism and cellular redox functions. (**A**) Western blot analysis of PHGDH and PSAT1 upon XRCC1 KD. α-Tubulin served as a loading control. (**B**) Quantifications of protein levels as seen in (**A**). Values are mean ± SD from eight independent experiments, normalized to the loading control. The *P*-values were calculated using Student's *t*-test. (**C**) mRNA levels of PHGDH and PSAT1 after XRCC1 KD, quantified by qPCR and normalized to GAPDH. Values are mean ± SD from 10 independent experiments. The *P*-values were calculated using Student's *t*-test. (**D**) mRNA levels of ATF4 after XRCC1 KD, quantified by qPCR and normalized to GAPDH. Values are mean ± SD from three independent experiments. The *P*-value was calculated using Student's *t*-test. (**E**) Western blot analysis of PHGDH and PSAT1 upon KD of ATF4, XRCC1 or both. β-Actin served as a loading control. (**F**) mRNA levels of ATF4 and PSAT1 after KD of ATF4, XRCC1, or both, quantified by qPCR and normalized to GAPDH. Values are mean ± SD from two independent experiments. (**G**) Western blot analysis of PHGDH and PSAT1 upon KD of XRCC1 in G1 arrested cells. α-Tubulin served as a loading control.

**Figure 4.**
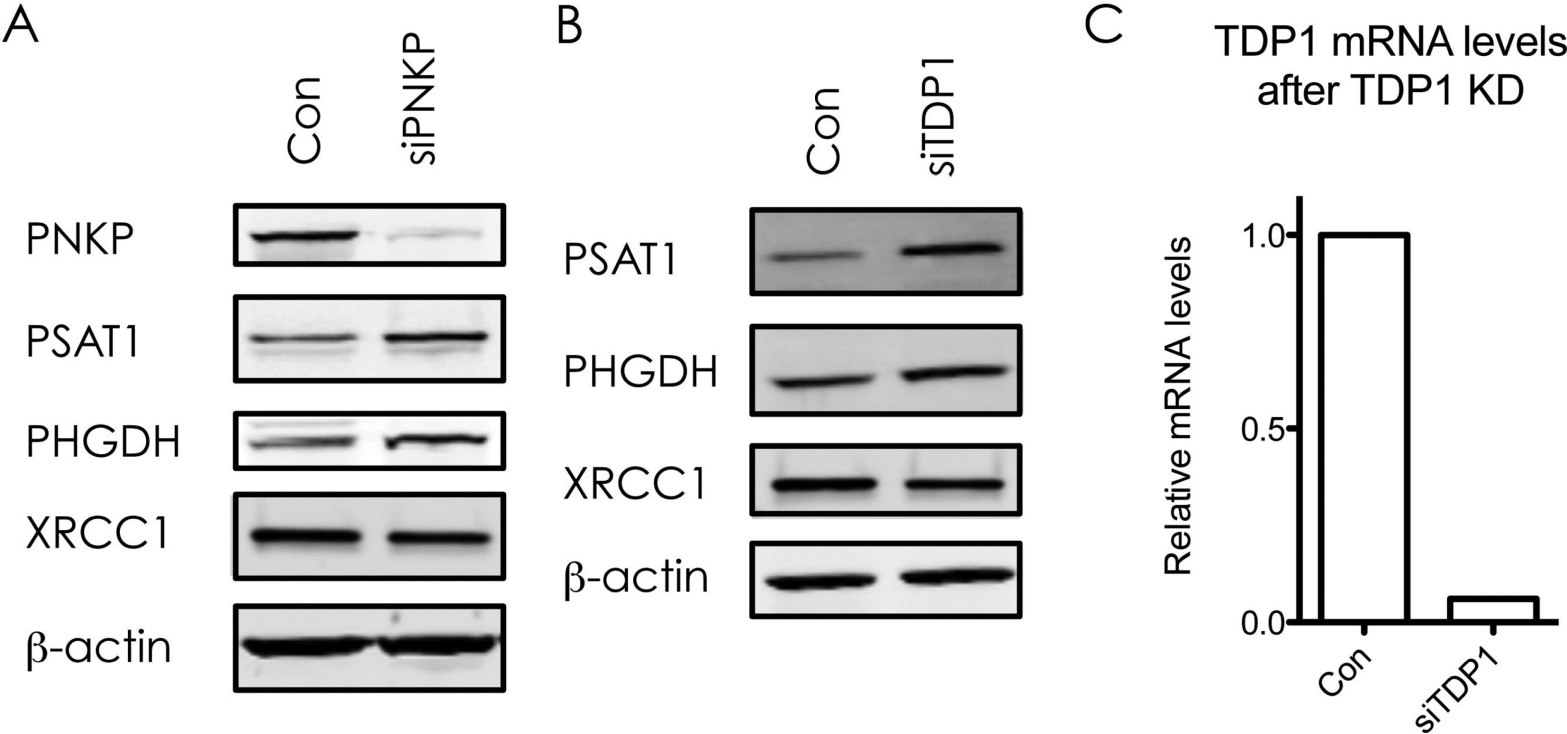
Knockdown of PNKP or TDP1 leads to increase in expression of PSAT1 and PHGDH. Western blot analysis of XRCC1, PHGDH and PSAT1 expression upon KD of PNKP (**A**) or TDP1 (**B**). β**-**Actin served as a loading control. (**C**) TDP1 mRNA levels after KD of TDP1, quantified by qPCR and normalised to GAPDH.

Metabolic reprogramming of cancer cells was first proposed by Otto Warburg (reviewed (21)), and is one of the hallmarks of cancer (2). One of the central metabolic pathways for cellular homeostasis is one-carbon metabolism, which encompasses the serine biosynthesis pathway, and, over the last few years, has come to be recognized to play a pivotal role in tumorigenesis (22). Because of its importance for cellular metabolism, we were highly intrigued by the fact that the one carbon metabolism scored among the most significantly deregulated pathways in our heatmap analysis (Table 1, blue). More specifically, XRCC1 KD cells displayed a significant increase in the three key enzymes involved in serine biosynthesis, PHGDH, PSAT1 and PSPH (Table 3, blue). The upregulation of PSAT1 and PHGDH upon XRCC1 KD was further validated by western blot as well as on mRNA level by qPCR (Figure 3A–C), and with a second siRNA sequence against XRCC1 (Supplementary Figure S1). Transcription of PHGDH, PSAT1 and PSPH has been shown to be regulated by ATF4 (23). ATF4 is a transcription factor that induces the expression of a variety of anabolic genes involved in nutrient uptake, amino acid metabolism, and anti-oxidation in response to a variety of different stresses, in a process termed integrative stress response (ISR) (24). Consistent with this, we found an increase in ATF4 mRNA after XRCC1 KD, suggesting the activation of the ISR upon XRCC1 KD (Figure 3D). This was validated with a second siRNA sequence against XRCC1 (Supplementary Figure S1E). To verify the causal role of ATF4 in the upregulation of the one-serine metabolism upon XRCC1 KD, we performed a combined KD of ATF4 and XRCC1. Indeed, KD of ATF4 completely abrogated the XRCC1-KD mediated upregulation of PSAT1 and PHGDH on protein as well as on mRNA levels (Figure 3E and F). To exclude that the observed upregulation of the enzymes involved in one-carbon metabolism was merely a result of the G1 arrest, we performed a KD of XRCC1 in G1 arrested cells. Similarly to the non-synchronized cells, KD of XRCC1 in G1 arrested cells also led to an increase of PSAT1 and PHGDH, ruling out an indirect cell-cycle mediated effect (Figure 3G). To demonstrate that the observed changes in gene expression of PSAT1 and PHGDH are due to accumulation of DNA SSBs and are not caused by some side effects of XRCC1 KD, we investigated the effect of PNKP and TDP1 knockdown on the expression of PSAT1 and PHGDH. Both PNKP and TDP1 are required for processing of damaged DNA strand breaks and a deficiency in either of these proteins leads to accumulation of SSBs (25). We found that, similar to XRCC1 knockdown, KD of either PNKP or TDP1 lead to an increased expression of both PSAT1 and PHGDH (Figure 4A–C). Importantly, XRCC1 levels did not changed after either PNKP or TDP1 knockdown. These results clearly demonstrated that the observed upregulation of the one-carbon metabolism upon accumulation of persistent DNA single strand breaks is activated by BER deficiency and is due to an activation of the integrated stress response pathway governed by ATF4.

**Figure 5.**
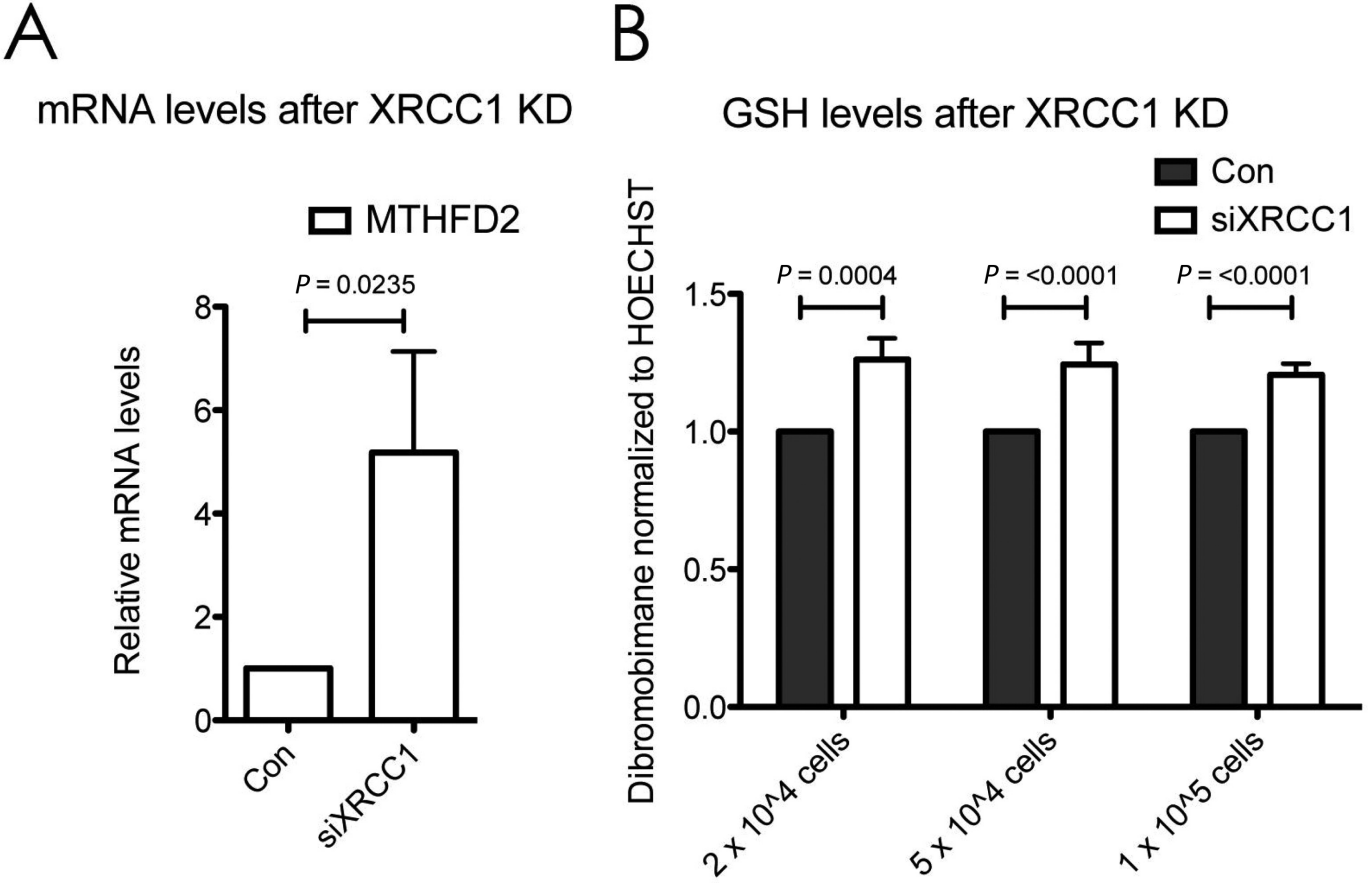
Increased expression of serine biosynthesis pathway enzymes after XRCC1 knockdown. (**A**) mRNA levels of MTHFD2 after XRCC1 KD, quantified by qPCR and normalised to GAPDH. Values are mean ± SD from four independent experiments. The *P*-value was calculated using Student's *t*-test. (**B**) Measurement of GSH levels in cells using fluorescence of Dibromobimane normalised to fluorescence of Hoechst 33342. Control or XRCC1 KD cells were seeded 24 h prior to measurement at the indicated densities into 24-well plates. Values are mean ± SD from three independent experiments with four replicates each. The *P*-values were calculated using Student's *t*-test.

The serine biosynthesis pathway (Supplementary Figure S2) provides direct inputs into the one-carbon metabolism, which consists of the folate and the methionine cycles, as well as the transsulfuration pathway. By donating a carbon unit from its side chain to tetrahydrofolate, serine feeds directly into the folate cycle. The folate cycle can provide building blocks for nucleotide synthesis, and additionally fuels the methionine cycle and the transsulfuration pathway. As a ‘side product’ of the carbon donation step, serine itself is converted to glycine, which in turn can again directly feed the folate cycle. Interestingly, the tetrahydrofolate salvage pathway involving the enzyme MTHFD2 was significantly upregulated upon XRCC1 KD both on protein (Table 1, rank 12) and mRNA levels (Figure 5A), suggesting that this metabolic branch is indeed in a state of enhanced activity. This was verified with a second siRNA sequence against XRCC1 (Supplementary Figure S1). Of note, ATF4^−/-^ cells have been found to display decreased levels of MTHFD2 (24), suggesting that MTHFD2 up-regulation in XRCC1 KD cells may be a consequence of increased ATF4 expression observed in our experiments (Figure 3D). In addition to the increase in intracellular synthesis of amino acids by the serine biosynthesis pathway, we also observed an increase in expression of a variety of amino acid transporters of the solute carrier family (Table 3, light red). Interestingly, SLC1A4, SLC3A2, SLC7A1 and SLC7A5 are all regulated by ATF4 as well (24,26). These changes further support the notion that XRCC1 KD promotes changes in cellular metabolism toward an anabolic state via the ATF4-mediated ISR pathway.

One of the outputs of serine metabolism is to contribute to the biosynthesis of glutathione (GSH), a major cellular antioxidant, which can scavenge reactive oxygen species and contribute to the maintenance of the appropriate NADPH/NADP^+^ ratios for cellular metabolism. GSH is a tripeptide, consisting of cysteine, glycine and glutamate. By condensing with homocysteine from the methionine cycle, serine is converted to GSH via intermediate steps by the transsulfuration pathway (22). Consistent with an activation of the one-carbon metabolism, and specifically also the transsulfuration pathway, we observed a clear increase in intracellular GSH levels upon XRCC1 KD (Figure 5B). Interestingly, enzymes involved in antioxidant functions (such as glutathione peroxidase 1, microsomal glutathione S-transferase 2, FOS-like antigen 1 and heme oxygenase (decycling) 1) were also differentially regulated upon XRCC1 KD (Table 3, light purple), suggesting the activation of a cellular redox-stress response.

In summary, these findings imply that cells with persistent SSBs activate a stress-response involving serine biosynthesis and the one-carbon metabolism, as well as amino acid uptake, thus instituting a change toward an anabolic cellular state by enhancing their amino acid synthesis as well as an increased antioxidant capacity via the production of GSH.

### Energy metabolism

A recently proposed concept by Maddocks *et al*. suggested that an increased activity in the serine biosynthesis pathway in cancer cells leads to a decrease in pyruvate levels due to the metabolic rerouting of glycolytic intermediates (27). To compensate for the resulting decrease in pyruvate levels, cells preferentially metabolised the remaining pyruvate via the more energy-efficient TCA cycle, in order to generate sufficient amounts of ATP for cellular transactions via mitochondrial respiration. In agreement with this idea, our data showed a clear increase in various components of the mitochondrial respiratory chain, including ATP synthase coupling factor 6, carnitine palmitoyltransferase 1A, and NADH dehydrogenase Fe–S proteins 1 and 3 (Table 3, light green).

### XRCC1 knockdown leads to increased expression of genes linked to excessive tissue remodelling, cellular motility and invasiveness

The classical clinical presentation of a cancer as a ‘lump’ can be attributed to the so-called ‘desmoplastic reaction’ of tumor and surrounding tissue, which is characterised by an excessive deposition of collagens and other components of the extracellular matrix (ECM) (28). This reaction is similar to the stromal reaction to a wound—indeed, tumors have been likened to wounds, that do not heal (29). Likewise, fibrosis is a process that can result from incorrect wound-healing, and is associated with an excessive accumulation of ECM proteins due to activation of a cascade of pro-inflammatory events. Interestingly, our results showed that XRCC1 KD leads to a proteomics profile much reminiscent of inflammatory events in hepatic fibrosis (Table 1, rank 1). Detailed analysis of the involved proteins revealed significant increases in the production of collagens as well as tissue modulators, such as MMP2 and TIMP2 (Table 3, white). Closely associated pathways involving similar proteins (‘inhibition of matrix metalloproteases’, ‘intrinsic prothrombin activation pathway’, ‘atherosclerosis signalling’, ‘heme degradation’, ‘basal cell carcinoma signalling’, ‘Il-8 signalling’, ‘role of Wnt/GSK-3β signalling’ and ‘germ cell-sertoli cell junction signalling’) were also deregulated in XRCC1 KD cells (Tables 1 and 3, ‘tissue remodelling’). These events suggested the establishment of a phenotype of active ECM remodeling, a known feature of malignancy (30), by cells that harbour genetic instability in form of persistent SSBs. Modulation of the ECM is also an important feature of the epithelial to mesenchymal transition (EMT) that forms part of the invasion and metastasis hallmark of cancer (2). Consistent with the notion that XRCC1 KD cells are modulating their intra- and extracellular morphology (above), also an enhancement of EMT-associated proteins, such as WNT5B, became evident in our dataset (Tables 1 and 3). Furthermore, many proteins associated with tissue remodelling and cellular invasion (e.g. TIMP1, TIMP2) were clearly deregulated in the dataset in a manner suggesting a heightened state of cellular plasticity (e.g. ‘glioma invasiveness signalling’) (Tables 1 and 3). An extension of analysis to pathways beyond the ones ranking in the top 17 revealed a multitude of other proteins involved in cellular motility and invasiveness, and associated with metastasis to be significantly deregulated (Table 3, all proteins with white background without associated pathway number in the last column).

In conclusion, the data presented here shows that the build-up of endogenous DNA lesions brought about by a deficiency in BER capacity can provoke gene expression changes that lead to changes in a variety of cellular pathways in primary cells very much reminiscent of changes that are hallmarks of cancerous cells. Furthermore, our results suggest that changes in the hallmarks of cellular metabolism as well as invasion and metastasis are not late-onset adaptations in cancer cells, but might be traits that are acquired at an early time-point due to genetic instability. Therefore, we find that a deficiency in BER can rewire cellular metabolism toward a cancer-like phenotype even in the absence of exogenous DNA damage, thus underpinning the importance of BER of endogenous DNA damage.

## DISCUSSION

The DNA damage response, which is clearly detected to be activated by XRCC1 KD, activates many proteins including transcription factors such as p53, E2F1 and Sp1 as well as a subset of protein kinases, including ATM and AKT (reviewed in e.g. (4,31)). All these proteins induce a cascade of changes that in turn will affect gene expression spectrum bringing cellular metabolism in terms with a new intracellular landscape caused by genetic instability. Importantly, however, there is a crucial temporal difference between the classical DNA damage response and the changes we observe upon XRCC1 KD. Classically, the canonical DNA damage response is a mechanism to respond to acute DNA damage and results in short-term adaptations in the cell, which are reverted upon disappearance of the harmful stimulus and repair of the damage. In contrast to this, XRCC1 KD cells are dealing with a long-term activation of the DNA damage response (in this study, it was lasting for several days). Thus, the changes we observe reflect long-term adaptive mechanisms that are evoked by the inability of cells to return to a basal, undisturbed state for long enough to completely revert the activation of the damage response. We hypothesise that this leads to an effect akin of ‘cellular exhaustion’, which ultimately results in the reprogramming of cells gene expression. Notably, these changes are a ‘signature’ of genetic instability that can provoke cell transformation by generating new mutations, and this ‘signature’ possibly remains present even in cancer cells. This hypothesis is in clear correlation with our detailed analysis of the data, which demonstrated significant changes in the XRCC1 KD cells toward a *phenotype detected in cancer cells*. Importantly, a big proportion of the significantly deregulated targets in our study have been reported as deregulated in a similar manner in human cancers as well (Supplementary Material, Supplementary Table S1). Interestingly, the only targets clearly counter-regulated and thus inhibiting further proliferation were the proteins associated with DNA replication itself, suggesting that the G_1_-S checkpoint was still a functional brake protecting these cells from true malignant transformation. Taken together, these results suggest that XRCC1 knockdown causes pathway changes that correlate strongly with a ‘cancer blueprint’.

Analysis of available epidemiological literature on cancer incidence and its associations with alterations in DNA repair pathways support the involvement of BER, and in particular XRCC1, in cancer risk. So far, most epidemiological research concerning XRCC1 has been focusing on the prevalence of polymorphisms, and possible links to cancer risk for carriers of certain polymorphic traits. Unfortunately however, only an absolute minority of these studies have considered analysing the levels of XRCC1 protein in relation to these polymorphisms, which might be a very important factor considering that XRCC1 has no enzymatic activity but functions to coordinate the steady-state levels and the activities of BER proteins as a molecular scaffold. Nevertheless, one study did show that a novel polymorphism in XRCC1 gene reduced its promoter activity, thus leading to lower protein levels and increased risk of non-small cell lung cancer (32). There are some studies however, which have directly addressed the expression levels of XRCC1 in normal versus tumour tissue. Interestingly, XRCC1 has been found to be downregulated in a number of different tumors (33–35). Decreased expression was associated with higher proliferation (and thus increased malignancy), as judged by Ki-67 staining (36), and shorter overall survival (34). A dysregulation of XRCC1, among a number of other targets, was found to be associated with higher prevalence of lung cancer (37). The relative expression of XRCC1 was significantly lower in peripheral blood mononuclear cells from esophageal squamous cell carcinoma patients compared to the control group (38). Additionally, CpG island hypermethylation of XRCC1 was found to increase gradually throughout lung carcinogenesis in a chemical-induced rat carcinogenesis model. Also, a decrease in XRCC1, and thus defective BER, seems to be a contributing factor to the development of myeodysplastic and myeloproliferative diseases (39). Similarly, lower expression levels of XRCC1 in bladder cancer tumour cells were associated with worse survival after radiotherapy (40). All of these observations support the conclusion from our study, that a deficiency in BER can create a favourable condition for malignant transformation of cells.

## SUPPLEMENTARY DATA

Supplementary Data are available at NAR Online.

SUPPLEMENTARY DATA
