# Supplementary material for: Cells deficient in base-excision repair reveal cancer hallmarks originating from adjustments to genetic instability
Source: Nucleic Acids Res. 2015 Mar 23;43(7):3667–79. doi: 10.1093/nar/gkv222 (PMC4402536; doi:10.1093/nar/gkv222)
Supplement: SUPPLEMENTARY DATA [file supp_43_7_3667__index.html]

Cells deficient in base-excision repair reveal cancer hallmarks originating from adjustments to genetic instability — Cells deficient in base-excision repair reveal cancer hallmarks originating from adjustments to genetic instability — SUPPLEMENTARY DATA 

# Cells deficient in base-excision repair reveal cancer hallmarks originating from adjustments to genetic instability

## SUPPLEMENTARY DATA

**Files in this Data Supplement:**

- SUPPLEMENTARY DATA
- SUPPLEMENTARY DATA
- SUPPLEMENTARY DATA
- SUPPLEMENTARY DATA
