# Supplementary figures and images for: Cells deficient in base-excision repair reveal cancer hallmarks originating from adjustments to genetic instability
Source: Nucleic Acids Res. 2015 Mar 23;43(7):3667–79. doi: 10.1093/nar/gkv222 (PMC4402536; doi:10.1093/nar/gkv222)

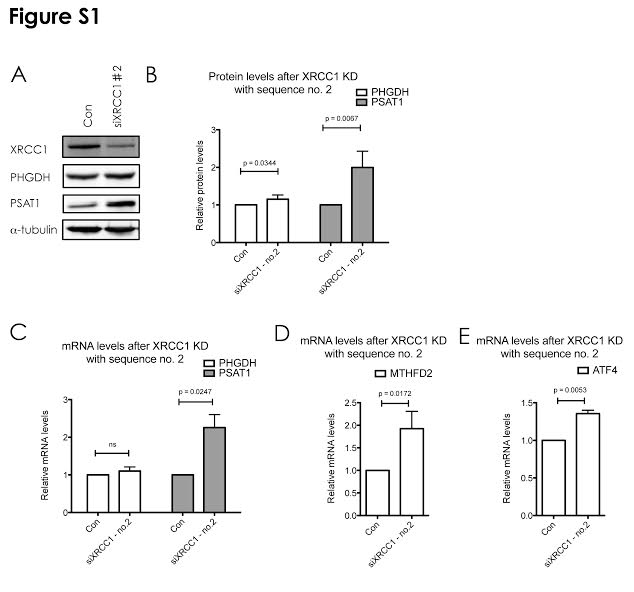

Supplement: SUPPLEMENTARY DATA [file supp_gkv222_nar-00221-d-2015-File007.jpg]

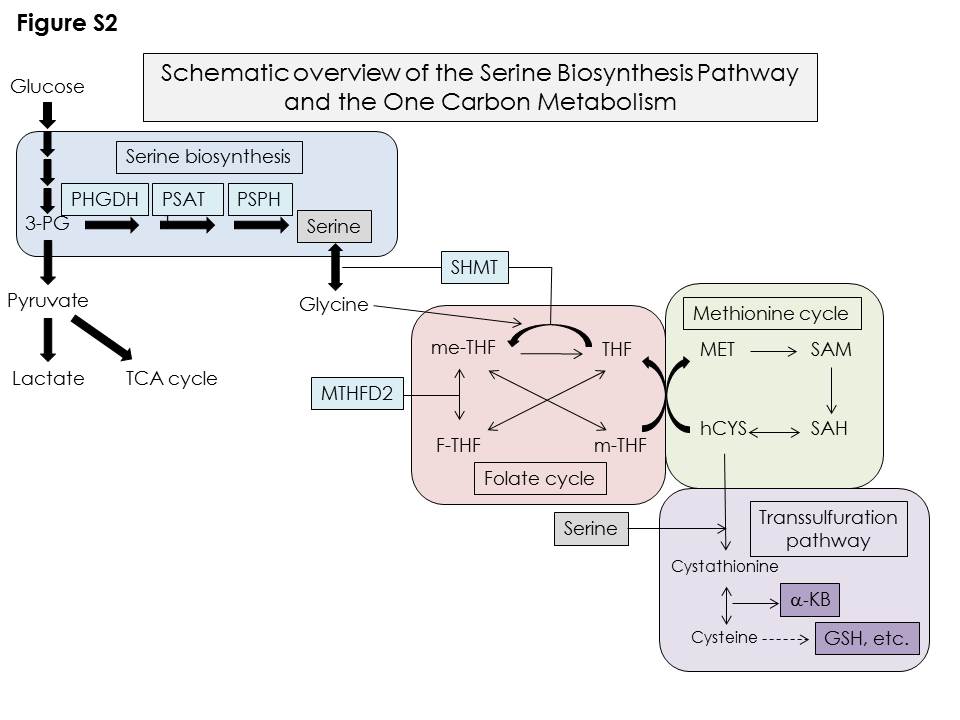

Supplement: SUPPLEMENTARY DATA [file supp_gkv222_nar-00221-d-2015-File008.jpg]
