# Supplementary material for: Cells deficient in base-excision repair reveal cancer hallmarks originating from adjustments to genetic instability
Source: Nucleic Acids Res. 2015 Mar 23;43(7):3667–79. doi: 10.1093/nar/gkv222 (PMC4402536; doi:10.1093/nar/gkv222)
Supplement: SUPPLEMENTARY DATA [file supp_gkv222_nar-00221-d-2015-File009.docx]

**Table S1. Changes in the key proteins found deregulated in XRCC1 KD cells are reflecting changes found in cancer samples.**

Overview of the deregulated proteins found in DNA repair deficient (XRCC1 KD) cells that are also deregulated in a similar manner in cancer cells. The first column indicates the pathways involved, the second column specifies the single proteins that were significantly changed upon XRCC1 KD, while the third column lists the change observed in cancer cells. 🡹 stands for proteins that are upregulated, 🡻 stands for proteins that are downregulated. Only proteins found to be significantly deregulated in the XRCC1 KD dataset whose upregulation clearly correlates with carcinogenesis are listed; For proteins with abundant data only selected papers are cited. Proteins shown in the table were chosen according to following criteria: (1) Among the top hits of the combined pathway analysis with a p-value *≤ 0.01*, **or** pathways closely related to the former ones with proteins upregulated ≥ 2 –fold in the MS/MS analysis; (2) Shown are only proteins/pathways whose upregulation/increased expression has been demonstrated to clearly correlate with cancer or cause malignant transformation

**Supplementary Table S1: Changes in the key proteins found deregulated in XRCC1 KD cells are reflecting changes found in cancer samples.**

| Highly deregulated pathways in DNA repair deficient cells (XRCC1 kd) that are **correlated** to carcinogenesis | Changed protein in XRCC1 KD cells | Changes observed in cancer cells,  Involvement in cancer |
| --- | --- | --- |
| Tissue remodeling  Epithelial to mesenchymal transition  Cellular invasion  Cell movement | COL1A1 🡹, COL1A2 🡹, COL3A1 🡹 | 🡹 e.g. (1,2), reviewed in (3,4) |
|  | MMP2 🡹 | 🡹 (5), and many more reviewed in (6) |
|  | MYH11 🡻 | 🡻 (7) |
|  | TIMP2 🡹 | Activates MMP2, Reviewed in (6) |
|  | ITGB5 🡹 | 🡹 (8) |
|  | HMOX1 🡹 | 🡹 Reviewed in (9) |
|  | FZD2 🡹 | 🡹 (10,11) |
|  | WNT5B 🡹 | 🡹 (12-14) |
|  | DVL3 🡹 | 🡹 (15,16) and others |
|  | RHOG 🡹 | 🡹 (17) |
|  | FOSL1 🡹 | 🡹 (18,19), reviewed in (20) |
|  | NRP2 🡹 | 🡹 Reviewed in (21) |
|  | PALLD 🡹 | 🡹 (22,23) and others |
|  | ICAM1 🡹 | 🡹 reviewed in (24) |
| Serine biosynthesis  (One-carbon metabolism) | PHGDH 🡹 | 🡹 (25-29) |
|  | PSAT1 🡹 | 🡹 (25-29) |
|  | PSPH 🡹 | 🡹 (25-29) |
| Tetrahydrofolate cycle  (One-carbon metabolism) | MTHFD2 🡹 | 🡹 (30-34) |
|  | SHMT2 🡹 (<2-fold) | 🡹 (30,32) |
| Pentose phosphate pathway  (Nucleotide synthesis) | G6PD 🡹 (<2-fold) | 🡹 (26) (35,36) and many more |
|  | PRPS1 🡹 |  |
| Amino acid metabolism | SLC3A2 🡹 | 🡹 (37) (38) |
|  | SLC7A5 🡹 | 🡹 (39-41), reviewed in (42) |
|  | SLC1A5 🡹 | 🡹 (43,44) and many more, reviewed in (42) |
|  | SLC7A11 🡹 | 🡹 (45,46), reviewed in (42) |

**References**

1. Kauppila S, Stenbäck F, Risteli J, Jukkola A, Risteli L. Aberrant type I and type III collagen gene expression in human breast cancer in vivo. J Pathol. 1998;186:262–8.

2. Pepin F, Bertos N, Laferriere J, Sadekova S, Souleimanova M, Zhao H, et al. Gene expression profiling of microdissected breast cancer microvasculature identifies distinct tumor vascular subtypes. Breast Cancer Res. 2012;14:R120.

3. Luparello C. Aspects of Collagen Changes in Breast Cancer. J Carcinogene Mutagene. 2013;S13.

4. Nerenberg PS, Salsas-Escat R, Stultz CM. Collagen--a necessary accomplice in the metastatic process. Cancer Genomics Proteomics. 2007;4:319–28.

5. Burduk PK, Bodnar M, Sawicki P, Szylberg L, Wiśniewska E, Kazmierczak W, et al. Expression of MMP2, MMP9, TIMP1 and TIMP2 could predict lymph node metastases in oropharyngeal squamous cell carcinoma. Head Neck. 2014.

6. Egeblad M, Werb Z. New functions for the matrix metalloproteinases in cancer progression. Nat Rev Cancer. 2002;2:161–74.

7. Kruhøffer M, Jensen JL, Laiho P, Dyrskjøt L, Salovaara R, Arango D, et al. Gene expression signatures for colorectal cancer microsatellite status and HNPCC. Br J Cancer. 2005;92:2240–8.

8. Bianchi-Smiraglia A, Paesante S, Bakin AV. Integrin β5 contributes to the tumorigenic potential of breast cancer cells through the Src-FAK and MEK-ERK signaling pathways. Oncogene. 2013;32:3049–58.

9. Jozkowicz A, Was H, Dulak J. Heme oxygenase-1 in tumors: is it a false friend? Antioxid Redox Signal. 2007;9:2099–117.

10. Wang Y, Zheng T. Screening of Hub Genes and Pathways in Colorectal Cancer with Microarray Technology. Pathol Oncol Res. 2014.

11. Salsano E, Paterra R, Figus M, Menghi F, Maderna E, Pollo B, et al. Expression profile of frizzled receptors in human medulloblastomas. J Neurooncol. 2012;106:271–80.

12. Kuorelahti A, Rulli S, Huhtaniemi I, Poutanen M. Human chorionic gonadotropin (hCG) up-regulates wnt5b and wnt7b in the mammary gland, and hCGbeta transgenic female mice present with mammary Gland tumors exhibiting characteristics of the Wnt/beta-catenin pathway activation. Endocrinology. 2007;148:3694–703.

13. Lu D, Zhao Y, Tawatao R, Cottam HB, Sen M, Leoni LM, et al. Activation of the Wnt signaling pathway in chronic lymphocytic leukemia. Proc Natl Acad Sci USA. 2004;101:3118–23.

14. Mangioni S, Viganò P, Lattuada D, Abbiati A, Vignali M, Di Blasio AM. Overexpression of the Wnt5b gene in leiomyoma cells: implications for a role of the Wnt signaling pathway in the uterine benign tumor. Journal of Clinical Endocrinology &amp; Metabolism. 2005;90:5349–55.

15. Kwan HT, Chan DW, Cai PCH, Mak CSL, Yung MMH, Leung THY, et al. AMPK activators suppress cervical cancer cell growth through inhibition of DVL3 mediated Wnt/β-catenin signaling activity. PLoS ONE. 2013;8:e53597.

16. Wei Q, Zhao Y, Yang Z-Q, Dong Q-Z, Dong X-J, Han Y, et al. Dishevelled family proteins are expressed in non-small cell lung cancer and function differentially on tumor progression. Lung Cancer. 2008;62:181–92.

17. Jiang WG, Watkins G, Lane J, Cunnick GH, Douglas-Jones A, Mokbel K, et al. Prognostic value of rho GTPases and rho guanine nucleotide dissociation inhibitors in human breast cancers. Clin Cancer Res. 2003;9:6432–40.

18. Pennanen PT, Sarvilinna NS, Toimela T, Ylikomi TJ. Inhibition of FOSL1 overexpression in antiestrogen-resistant MCF-7 cells decreases cell growth and increases vacuolization and cell death. Steroids. 2011;76:1063–8.

19. Chiappetta G, Ferraro A, Botti G, Monaco M, Pasquinelli R, Vuttariello E, et al. FRA-1 protein overexpression is a feature of hyperplastic and neoplastic breast disorders. BMC Cancer. 2007;7:17.

20. Young MR, Colburn NH. Fra-1 a target for cancer prevention or intervention. Gene. 2006;379:1–11.

21. Prud'homme GJ, Glinka Y. Neuropilins are multifunctional coreceptors involved in tumor initiation, growth, metastasis and immunity. Oncotarget. 2012;3:921–39.

22. Pogue-Geile KL, Chen R, Bronner MP, Crnogorac-Jurcevic T, Moyes KW, Dowen S, et al. Palladin mutation causes familial pancreatic cancer and suggests a new cancer mechanism. PLoS Med. 2006;3:e516.

23. Goicoechea SM, Bednarski B, Stack C, Cowan DW, Volmar K, Thorne L, et al. Isoform-specific upregulation of palladin in human and murine pancreas tumors. PLoS ONE. 2010;5:e10347.

24. Roland CL, Harken AH, Sarr MG, Barnett CC. ICAM-1 expression determines malignant potential of cancer. Surgery. 2007;141:705–7.

25. Possemato R, Marks KM, Shaul YD, Pacold ME, Kim D, Birsoy K, et al. Functional genomics reveal that the serine synthesis pathway is essential in breast cancer. Nature. 2011;476:346–50.

26. Hu J, Locasale JW, Bielas JH, O'Sullivan J, Sheahan K, Cantley LC, et al. Heterogeneity of tumor-induced gene expression changes in the human metabolic network. Nature Biotechnology. 2013;31:522–9.

27. Pollari S, Käkönen S-M, Edgren H, Wolf M, Kohonen P, Sara H, et al. Enhanced serine production by bone metastatic breast cancer cells stimulates osteoclastogenesis. Breast Cancer Res Treat. 2011;125:421–30.

28. Locasale JW, Grassian AR, Melman T, Lyssiotis CA, Mattaini KR, Bass AJ, et al. Phosphoglycerate dehydrogenase diverts glycolytic flux and contributes to oncogenesis. Nat Genet. 2011;43:869–74.

29. Tedeschi PM, Markert EK, Gounder M, Lin H, Dvorzhinski D, Dolfi SC, et al. Contribution of serine, folate and glycine metabolism to the ATP, NADPH and purine requirements of cancer cells. Cell Death Dis. 2013;4:e877.

30. Nilsson R, Jain M, Madhusudhan N, Sheppard NG, Strittmatter L, Kampf C, et al. Metabolic enzyme expression highlights a key role for MTHFD2 and the mitochondrial folate pathway in cancer. Nat Commun. 2014;5:3128.

31. Selcuklu SD, Donoghue MTA, Rehmet K, de Souza Gomes M, Fort A, Kovvuru P, et al. MicroRNA-9 inhibition of cell proliferation and identification of novel miR-9 targets by transcriptome profiling in breast cancer cells. Journal of Biological Chemistry. 2012;287:29516–28.

32. Jain M, Nilsson R, Sharma S, Madhusudhan N, Kitami T, Souza AL, et al. Metabolite profiling identifies a key role for glycine in rapid cancer cell proliferation. Science. 2012;336:1040–4.

33. Lehtinen L, Ketola K, Mäkelä R, Mpindi J-P, Viitala M, Kallioniemi O, et al. High-throughput RNAi screening for novel modulators of vimentin expression identifies MTHFD2 as a regulator of breast cancer cell migration and invasion. Oncotarget. 2013;4:48–63.

34. Xu X, Qiao M, Zhang Y, Jiang Y, Wei P, Yao J, et al. Quantitative proteomics study of breast cancer cell lines isolated from a single patient: discovery of TIMM17A as a marker for breast cancer. Proteomics. 2010;10:1374–90.

35. Du W, Jiang P, Mancuso A, Stonestrom A, Brewer MD, Minn AJ, et al. TAp73 enhances the pentose phosphate pathway and supports cell proliferation. Nature Publishing Group. 2013;15:991–1000.

36. Wang J, Yuan W, Chen Z, Wu S, Chen J, Ge J, et al. Overexpression of G6PD is associated with poor clinical outcome in gastric cancer. Tumour Biol. 2012;33:95–101.

37. Poettler M, Unseld M, Braemswig K, Haitel A, Zielinski CC, Prager GW. CD98hc (SLC3A2) drives integrin-dependent renal cancer cell behavior. Molecular Cancer. 2013;12:169.

38. Yang Y, Toy W, Choong LY, Hou P, Ashktorab H, Smoot DT, et al. Discovery of SLC3A2 cell membrane protein as a potential gastric cancer biomarker: implications in molecular imaging. J Proteome Res. 2012;11:5736–47.

39. Kaira K, Oriuchi N, Imai H, Shimizu K, Yanagitani N, Sunaga N, et al. Prognostic significance of L-type amino acid transporter 1 expression in resectable stage I-III nonsmall cell lung cancer. Br J Cancer. 2008;98:742–8.

40. Kobayashi K, Ohnishi A, Promsuk J, Shimizu S, Kanai Y, Shiokawa Y, et al. Enhanced tumor growth elicited by L-type amino acid transporter 1 in human malignant glioma cells. Neurosurgery. 2008;62:493–503–discussion503–4.

41. Kaira K, Oriuchi N, Imai H, Shimizu K, Yanagitani N, Sunaga N, et al. l-type amino acid transporter 1 and CD98 expression in primary and metastatic sites of human neoplasms. Cancer Sci. 2008;99:2380–6.

42. Nakanishi T, Tamai I. Solute carrier transporters as targets for drug delivery and pharmacological intervention for chemotherapy. J Pharm Sci. 2011;100:3731–50.

43. Kikuchi T, Hassanein M, Amann JM, Liu Q, Slebos RJC, Rahman SMJ, et al. In-depth proteomic analysis of nonsmall cell lung cancer to discover molecular targets and candidate biomarkers. Mol Cell Proteomics. 2012;11:916–32.

44. Hassanein M, Hoeksema MD, Shiota M, Qian J, Harris BK, Chen H, et al. SLC1A5 mediates glutamine transport required for lung cancer cell growth and survival. Clin Cancer Res. 2013;19:560–70.

45. Drayton RM, Dudziec E, Peter S, Bertz S, Hartmann A, Bryant HE, et al. Reduced expression of microRNA-27a modulates cisplatin resistance in bladder cancer by targeting the cystine/glutamate exchanger SLC7A11. Clin Cancer Res. 2014.

46. Liu X-X, Li X-J, Zhang B, Liang Y-J, Zhou C-X, Cao D-X, et al. MicroRNA-26b is underexpressed in human breast cancer and induces cell apoptosis by targeting SLC7A11. FEBS Lett. 2011;585:1363–7.
